# Timeliness of yellow fever specimen collection and transport in Ghana, 2018–2022

**DOI:** 10.1371/journal.pgph.0005703

**Published:** 2025-12-19

**Authors:** Seth D. Judson, Lee Schroeder, Franklin Asiedu-Bekoe, Dennis Odai Laryea, Gifty Boateng, Horlali Gudjinu, Robert Ossom, Jerry Fosu Danquah, David W. Dowdy, Ernest Kenu

**Affiliations:** 1 Department of Medicine, Johns Hopkins University School of Medicine, Baltimore, Maryland, United States of America; 2 Department of Medicine, David Geffen School of Medicine at UCLA, Los Angeles, California, United States of America; 3 Department of Pathology, University of Michigan, Ann Arbor, Michigan, United States of America; 4 Disease Surveillance Department, Public Health Division, Ghana Health Service, Accra, Ghana; 5 National Public Health and Reference Laboratory, Ghana Health Service, Accra, Ghana; 6 Department of Epidemiology, Johns Hopkins Bloomberg School of Public Health, Baltimore, Maryland, United States of America; 7 Department of Epidemiology, University of Ghana School of Public Health, Accra, Ghana; PLOS: Public Library of Science, UNITED STATES OF AMERICA

## Abstract

Yellow fever is a mosquito-borne viral hemorrhagic fever, and recent outbreaks of yellow fever have occurred in multiple African countries, including Ghana (2021–2022). Delayed diagnosis of yellow fever may cause increased morbidity and mortality. To improve timely detection of yellow fever, we need to better understand the factors contributing to diagnostic delays. We analyzed the diagnostic testing timeline of all suspected yellow fever cases in Ghana from 2018-2022. For these patients we calculated the days from symptom onset to specimen collection and arrival at the National Public Health and Reference Laboratory for testing. We compared these times to World Health Organization (WHO) metrics. For suspected yellow fever cases, the time from symptom onset to specimen arrival had a median of 10 days (interquartile range 6–17). 5892/6345 (93%) of specimens were collected within 14 days of symptom onset, and 2653/6471 (41%) of specimens arrived within 3 days of collection (WHO metrics). Overall, we find that the timing of yellow fever testing varies among districts in Ghana. While specimens are generally collected in a timely manner, delays in specimen arrival are common. Improving specimen transport for yellow fever and/or expanded testing could lead to more timely detection of outbreaks.

## Introduction

Multiple recent yellow fever (YF) outbreaks have occurred in Africa, and delays in diagnosing and implementing control measures for YF have been associated with increased morbidity and mortality [1,2]. It is estimated that approximately 100,000 severe YF cases occur in Africa annually on average [3]. While an effective vaccine for YF exists, there are ongoing gaps in immunization coverage [4]. Additionally, constraints on diagnostic resources and healthcare systems contribute to delayed detection of YF outbreaks [5].

Ghana is classified as a high-risk country for YF by the World Health Organization (WHO) and has had multiple YF outbreaks, including a recent YF outbreak from 2021-2022 [6–8]. Recent YF outbreaks in Ghana have originated in remote northern districts among unvaccinated populations that are far from the National Public Health and Reference Laboratory (NPHRL) that performs YF testing, potentially increasing the time to outbreak detection [6,9]. Early diagnosis of YF is important for supportive care and outbreak containment measures including vector control, risk communication, patient isolation, and reactive vaccination [2,4].

The WHO’s Eliminate Yellow Fever Epidemics (EYE) strategy was launched in 2017 in response to a YF epidemic in Africa that exhausted the global supply of YF vaccines [10]. A key part of the EYE strategy is accurate and timely laboratory diagnosis, following the WHO YF testing algorithm, which typically involves initial testing for YF IgM at national reference laboratories and confirmatory testing at regional reference laboratories [11,12]. A recent review of laboratory capacity for YF in 25 African countries found that lack of testing resources and shipping capacity limited confirmation of YF cases [13]. The WHO EYE strategy recommends that blood specimens are ideally collected within 14 days of symptom onset, so that reverse transcriptase polymerase chain reaction (RT-PCR) can be performed, leading to a faster confirmatory result [11]. In YF high-risk countries, the WHO also recommends specimens to be sent to the national reference laboratory within 3 days of collection for initial testing [11]. While the WHO has established an international YF specimen transport system for shipment to regional reference laboratories with funding from the Global Alliance for Vaccines and Immunization (GAVI) [14], countries must rely on their own transportation networks for national testing.

There are multiple steps in the YF diagnostic pathway that may contribute to delays in timely diagnosis. Patients in rural areas with limited access to healthcare resources may present late after symptom onset. There also could be delays in sample dispatch and transportation to national and regional reference laboratories. To better understand how different steps in the YF diagnostic pathway contribute to the timeliness of YF testing in Ghana, we evaluated the timing of symptom onset, specimen collection, and specimen arrival for testing for suspected YF cases in Ghana during 2018–2022 in relation to WHO metrics. The diagnostic pathway for YF in Ghana begins with YF IgM testing at the NPHRL, followed by confirmatory testing at the regional reference laboratory. Because of the time it takes for confirmatory testing, when there are multiple positive YF IgM results at the NPHRL in Ghana, an outbreak response is triggered. Additionally, the NPHRL in Ghana has recently gained the capacity for YF RT-PCR testing. Therefore, our analysis focused on the time between symptom onset and specimen arrival for initial YF testing, recognizing its critical role for timely case detection and outbreak response in Ghana.

## Methods

### Ethics statement

This study was approved as part of the Ghana Laboratory Network Project by the Ghana Health Service Ethical Review Committee (GHSERC: 005/05/19).

### Setting

The Republic of Ghana is a country in West Africa and part of the WHO Africa Region (AFRO) YF laboratory network. Since 2019, there have been 260 districts and 16 regions in Ghana. The districts and regions in Ghana for this study are shown in S1 Fig. One additional district (Guan) was added in October 2021 and is not included in this analysis. The northernmost regions in Ghana are where recent YF outbreaks have originated, with the Upper West, Savannah, and Upper East regions having the most reported YF cases since routine childhood immunization [6]. These regions are part of the Sudan Savannah and Guinea Savannah agro-climatic zones which are more arid and have more dispersed settlements compared to the southern zones [15], as well as more multidimensional poverty and less infrastructure [16]. Additionally these regions are closer to Mole National Park, the largest protected area in Ghana and a habitat for non-human primate species that are reservoir hosts for YF [6].

When a patient meets the suspected case definition for YF in Ghana (acute onset of fever followed by jaundice within the first two weeks of symptoms), a blood specimen must be collected and sent to the NPHRL for initial testing, usually consisting of a YF antibody capture enzyme-linked immunosorbent assay (MAC-ELISA) [11,17,18]. The NPHRL in Korle Bu has oversight over the zonal public health laboratories and does all YF IgM testing, forecasting, and resource procurement in Ghana. If there is a high index of suspicion for other viral hemorrhagic fevers, RT-PCR testing is done at the Noguchi Memorial Institute for Medical Research. Positive YF IgM specimens are transported to the WHO regional reference laboratory at Institut Pasteur de Dakar, Senegal for confirmatory testing.

### Data collection

The study population consisted of all suspected YF cases with blood specimens collected for testing in Ghana between January 1, 2018 and December 31, 2022. Data on these cases were collected from the Ghana Health Service’s Epi Info database. A limited data set was accessed for public health/research purposes on April 4, 2025. This data set included reporting health facility, patient’s town of residence and demographics, YF vaccination status, date of symptom onset, date patient was evaluated, date of specimen collection, date of specimen dispatch, date of specimen arrival at the NPHRL, and NPHRL test result. The exact dates of when the tests resulted at the NPHRL were unavailable. However, national protocols are that all YF testing at the NPHRL should be done within 72 hours of specimen arrival.

### Statistical analysis

Our primary outcomes of interest were overall days from symptom onset to specimen arrival at the NPHRL as well as (i) days from symptom onset to specimen collection and (ii) days from specimen collection to arrival at the NPHRL. Records with specimen collection and/or arrival dates occurring a year or more after symptom onset were excluded from our analysis due to suspected data entry errors (see S1 Appendix for cohort selection). We calculated national and district-level descriptive statistics for each of our outcomes. We compared these outcomes to two WHO metrics: the percent of specimens collected within 14 days of symptom onset and the percent of specimens that arrived at the NPHRL within 3 days of collection [11]. We also calculated these metrics pre and post COVID-19 (specimen collection during 2018–2019 and 2020–2022), given the influence the pandemic may have had on testing. Aggregate statistics by district and R scripts for the figures/analysis are available online [19]. All statistical analyses and figures were created using R version 4.4.0 [20].

We calculated Moran’s I to determine the degree of spatial autocorrelation among district-level YF testing statistics. District boundaries were obtained from the Ghana Statistical Service (GSS)/Humanitarian Data Exchange (https://data.humdata.org/dataset/cod-ab-gha). Moran’s I was calculated in R using the spdep package, and maps were created using the R packages: sf, ggplot2, viridis, and patchwork. A spatial weights matrix was constructed using queen contiguity and row-standardized weights were used with the assumption that spatial autocorrelation would be most influenced by immediate neighboring districts.

## Results

From 2018-2022, 6609 specimens were collected from 6447 patients to be tested for YF. The characteristics of these patients are shown in Table 1. The majority of tested patients were male (60%), lived in rural areas (74%), and were under 15 years of age (59%), with a median age of nine years (range 0–95 years). A record of receiving prior YF vaccination was available for 28% of patients, and the remaining had unrecorded vaccination status. Of the specimens with test results, 74/5142 (1%) were IgM positive for YF.

**Table 1:** Characteristics of patients tested for yellow fever in Ghana (2018–2022).

	Total (N = 6447)
**Age (years)**	
0-14	3809 (59.1%)
15-29	1406 (21.8%)
30-44	696 (10.8%)
45-59	227 (3.5%)
60+ Unknown	64 (1.0%) 245 (3.8%)
**Sex**	
F	2597 (40.3%)
M Unknown	3833 (59.5%) 17 (0.3%)
**Location of residence**	
Rural	4793 (74.3%)
Urban Unknown	1333 (20.7%) 321 (5.0%)
**Record of prior YF vaccination**	
Yes	1771 (27.5%)
No	4676 (72.5%)

**Fig 1:**
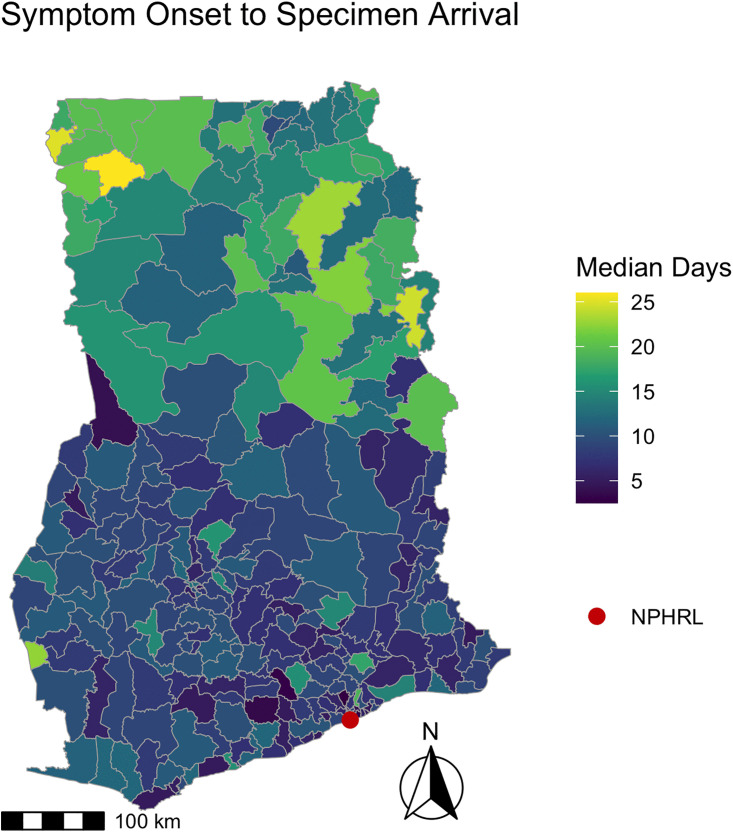
Time between symptom onset to specimen arrival for yellow fever testing by district in Ghana 2018-2022. For suspected YF cases from 2018-2022, the map depicts the median days for each district from symptom onset to specimen arrival at the NPHRL. The shapefile layers used to create the map were obtained from the Ghana Statistical Service (GSS)/Humanitarian Data Exchange (https://data.humdata.org/dataset/cod-ab-gha) and are publicly available under a CC BY-IGO license (https://data.humdata.org/faqs/licenses).

**Fig 2:**
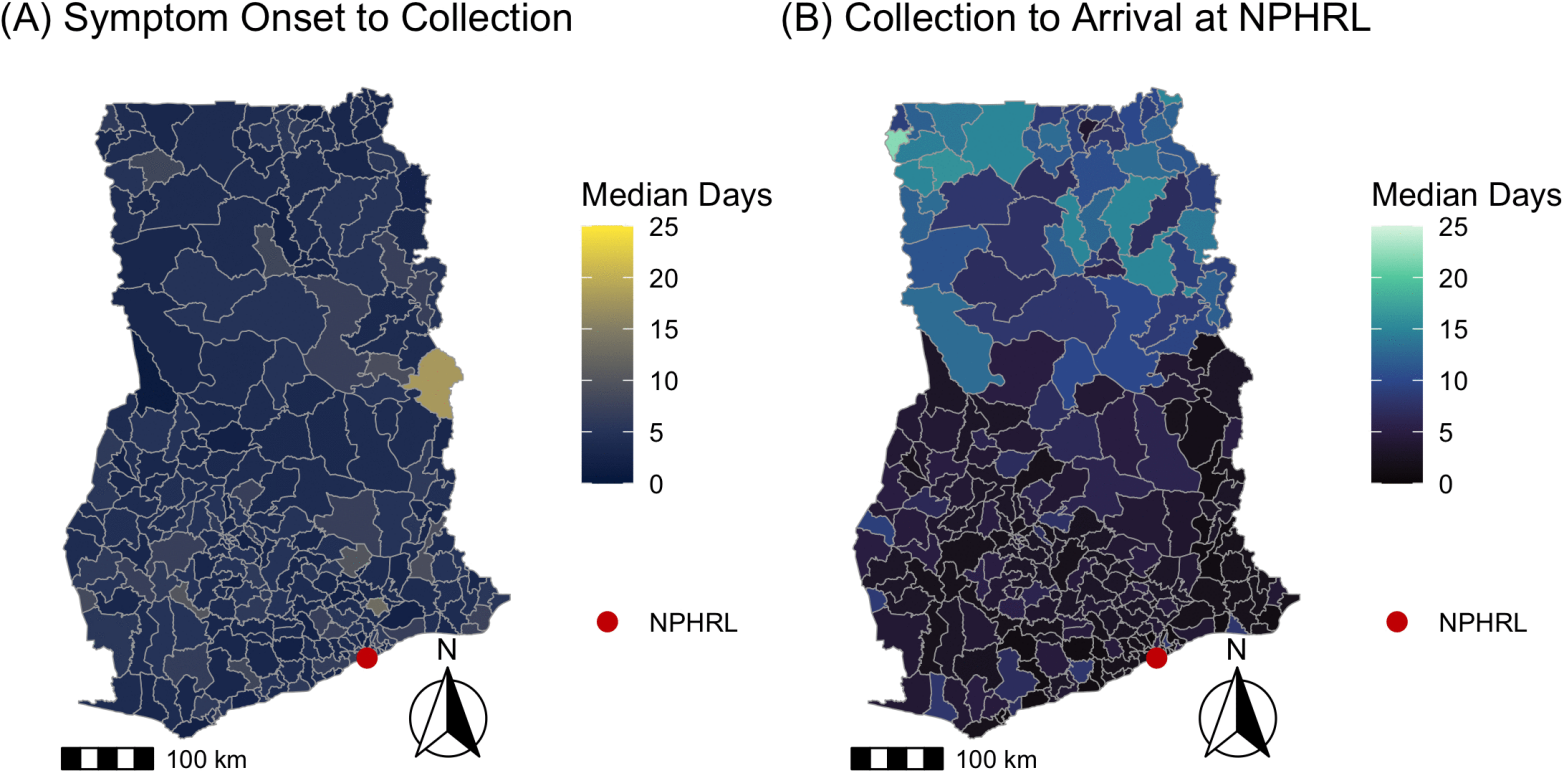
Timing of yellow fever specimen collection vs specimen arrival by district in Ghana 2018-2022. For suspected YF cases from 2018-2022, the maps depict the (A) median days from symptom onset to specimen collection vs (B) median days from specimen collection to specimen arrival at NPHRL. The shapefile layers used to create the maps were obtained from the Ghana Statistical Service (GSS)/Humanitarian Data Exchange (https://data.humdata.org/dataset/cod-ab-gha) and are publicly available under a CC BY-IGO license (https://data.humdata.org/faqs/licenses).

The median time from symptom onset to specimen arrival at the NPHRL was 10 days (IQR 6–17) overall and 11 days (IQR 7–15) for YF IgM positive cases. The majority of specimens (5892/6345, 93%) were collected within the WHO-recommended threshold of 14 days. The median time from symptom onset to specimen collection was 4 days (IQR 2–7 days), while the median time specimen collection to arrival at the NPHRL was 5 days (IQR 2–9 days). Less than half of specimens (2653/6471, 41%) arrived at the NPHRL within the WHO-recommended threshold of 3 days. Maps depicting the timing of YF specimen collection and arrival for testing are shown in Fig 1 and Fig 2.

Between 2018–2019 and 2020–2022, the proportion of specimens collected within 14 days of symptom onset remained at 93% (1936/2087 vs 3956/4258). However, the proportion of specimens arriving within 3 days of collection significantly decreased from 1097/2107 (52%) in 2018–2019 to 1556/4364 (36%) in 2020–2022 (two-sample Z test of proportions, p < 0.001). Full descriptive statistics comparing the entire study time period (2018–2022) to before and after COVID-19 (2018–2019 vs 2020–2022) are in the S1 Appendix.

**Fig 3:**
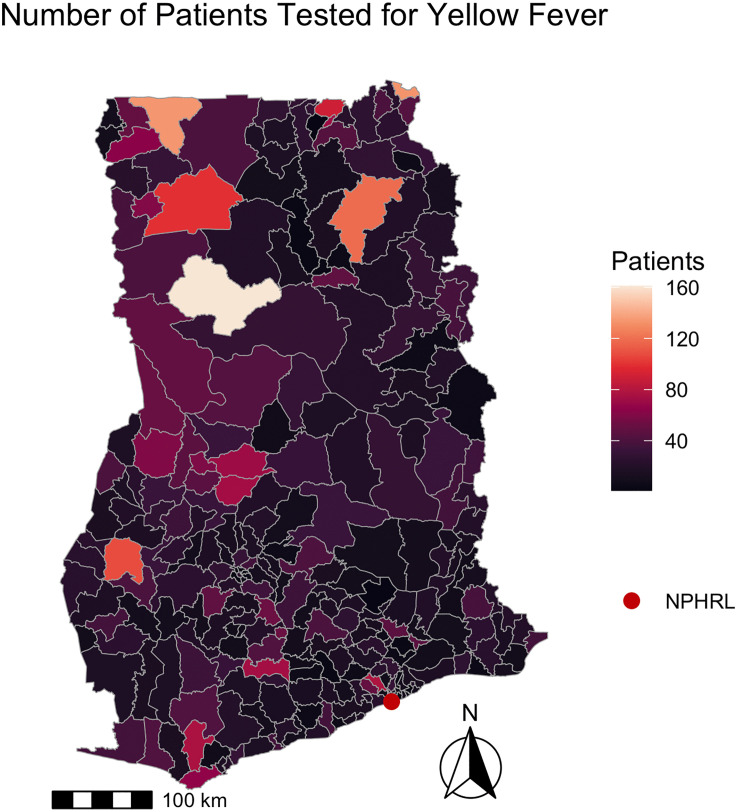
Number of patients tested for yellow fever by district in Ghana 2018-2022. The map depicts the number of patients tested for yellow fever by district from 2018-2022. The shapefile layers used to create the map were obtained from the Ghana Statistical Service (GSS)/Humanitarian Data Exchange (https://data.humdata.org/dataset/cod-ab-gha) and are publicly available under a CC BY-IGO license (https://data.humdata.org/faqs/licenses).

The West Gonja district, where the 2021–2022 YF outbreak originated, had the highest number of patients tested for YF (161). Fig 3 shows the number of patients tested for YF per district in 2018–2022.

There was significant spatial autocorrelation among districts regarding the number of patients tested (Moran’s I = 0.15, p < 0.001), the median days from symptom onset to specimen arrival (Moran’s I = 0.52, p < 0.001), and median days from specimen collection to arrival (Moran’s I = 0.69, p < 0.001). There was no significant spatial autocorrelation among districts regarding the median days from symptom onset to specimen collection (Moran’s I = 0.03, p = 0.22).

## Discussion

We found that the timing of specimen collection and arrival for YF testing varies across districts in Ghana. Nearly all samples (93%) in our study were collected within the WHO-recommended time frame of 14 days from symptom onset, during which RT-PCR can theoretically be performed. In contrast, we observed delays between specimen collection and arrival at the NPHRL for testing, with only 41% of specimens arriving within the WHO-recommended three days. These findings suggest targets for improving YF diagnosis at a country level in Ghana, specifically highlighting the importance of timely specimen transport.

Our observed duration (median four days) from symptom onset to specimen collection was similar to that seen in Uganda from 2017-2022 (median three days) [21]. In contrast, the time from symptom onset to specimen collection for YF contributed to 60% of the lag time in the Central African Republic from 2007-2012 [22] and was also higher during the 2016 YF outbreak in the Democratic Republic of the Congo (median 9 days) [2].

Our finding of frequent delays from specimen collection to arrival at NPHRL was also consistent with findings in Uganda [21], and likely reflects the structures of YF diagnostic networks in these countries. For example, in Ghana specimens from suspected YF cases are often batched prior to transportation to the NPHRL. This batching process likely contributes to the lag in specimen arrival for testing. Additionally, shortages of materials, challenges with cold chain, and unreliable transportation/road networks, especially in rural areas of Ghana, have been noted to affect specimen arrival for testing [23]. We also found that timely arrival of specimens to the NPHRL decreased by roughly a third during 2020–2022, which could reflect the impact of COVID-19 in straining the YF diagnostic network.

We also identified district-level patterns in the timeliness of YF testing in Ghana. Neighboring districts were more likely to have similar YF testing volume and time from specimen collection to arrival at the NPHRL. In all of the districts in the northernmost regions in Ghana (Upper West, Upper East, Savannah, Northern, and Northern East), the median time from specimen collection to arrival at the NPHRL was delayed beyond the WHO’s recommended threshold of within 3 days. This suggests that improving YF specimen transport or establishing testing closer to districts that are far from the NPHRL could improve timeliness of YF diagnosis and outbreak detection.

Given multiple recent outbreaks of viral hemorrhagic fevers, including YF, in Africa, there has been a recent emphasis on improving early detection and outbreak response [24–26]. Organizations such as the WHO and the Foundation for Innovative New Diagnostics (FIND) have assessed accessibility and optimizing diagnostic networks for infectious diseases [27]. The 7-1-7 strategy used by Resolve to Save Lives emphasizes outbreak detection within 7 days, public health notification within 1 day, and an initial response within 7 days [28]. These metrics as well as the WHO’s performance indicators can help guide timely YF diagnosis and response.

A limitation to our analysis is that we were unable to confirm the timing of when YF tests resulted or were reported to district health facilities. From interviews with NPHRL staff, we learned that there may be an inadequate supply of reagents for local YF IgM testing, and testing support from the WHO primarily occurs during outbreaks. Therefore, it is possible that resource constraints at the NPHRL may also contribute to delays in testing. We also learned that the confirmatory testing at the regional reference laboratory in Senegal could contribute further to diagnostic delays given the additional costs for transportation and prolonged turn-around time. We were unable to assess this part of the diagnostic pathway because confirmatory results on the IgM positive cases were not available. Furthermore, after an initial YF case is confirmed at the regional reference laboratory during an outbreak, subsequent IgM positive cases are often assumed to be cases and may not undergo further testing. Given that local public health responses for YF in Ghana have been initiated with clusters of YF IgM positivity prior to case confirmation and there is new capacity for YF RT-PCR at the NPHRL, the timing of initial testing in Ghana will be most important for local outbreak detection.

Our results suggest important targets for strengthening the YF diagnostic cascade in Ghana. Specifically, facilitating faster specimen transport could be prioritized. Ghana has national diagnostic transportation systems for HIV and tuberculosis [23], but combining these systems with YF has previously been challenging due to the differing network structures and specimen handling. Dried blood spots on FTA cards have been evaluated for YF and could enable faster transportation by avoiding the need for cold chain [29]. A pilot study in Mali showed that using a trained postal service improved timeliness for YF specimen arrival, however it also increased costs 8-fold [30]. Therefore, costs for a national YF transportation system in Ghana may be prohibitive, so it is important to consider alternative diagnostic strategies such as decentralized testing and point-of-care testing.

Considering decentralized testing, the Zonal Public Health Laboratory in Tamale is closer to the northern districts and could be an ideal location for expanded YF testing given recent outbreaks in this area [6]. Increasing capacity for YF testing at the northern Zonal lab could be an effective method for early outbreak detection. Furthermore, the timeliness of specimen collection suggests that RT-PCR could be performed, providing a faster confirmatory result. Alternatively, point-of-care tests, such as a lateral flow YF IgM assay that was recently validated in Ghana [31], could be used in remote districts. Such strategies could be especially important for timely case detection during outbreaks. For example, a mobile lab was deployed for YF testing during the 2016 YF epidemic in the Democratic Republic of the Congo with positive results [32]. While our study is specific to Ghana, our findings could be applicable to many other countries in Africa that have centralized testing for YF near urban capitals but have outbreaks originating in remote rural areas.

Analyzing national level diagnostic networks for YF will be essential for preparing and responding to future YF outbreaks. The timing of YF specimen arrival at the NPHRL in Ghana contributes to delays in testing, especially among more remote northern districts that could be at greater risk for YF outbreaks. Strengthening specimen transport systems and/or decentralized testing could improve timely detection of YF, leading to quicker patient isolation, supportive care, vector control, and vaccination campaigns.

## Supporting information

S1 Appendix Cohort selection and sensitivity analysis. (DOCX)

S1 Fig Ghana districts and regions 2018–2022. The 260 districts and 16 regions in Ghana that were analyzed for yellow fever testing from 2018-2022 are shown with the National Public Health and Reference Laboratory (NPHRL). The shapefile layers used to create the map were obtained from the Ghana Statistical Service (GSS)/Humanitarian Data Exchange (https://data.humdata.org/dataset/cod-ab-gha) and are publicly available under a CC BY-IGO license (https://data.humdata.org/faqs/licenses).
(DOCX)

## References

[1] Zhao S, Stone L, Gao D, He D. Modelling the large-scale yellow fever outbreak in Luanda, Angola, and the impact of vaccination. PLoS Negl Trop Dis. 2018;12(1):e0006158. doi: 10.1371/journal.pntd.0006158 29338001 PMC5798855

[2] Ingelbeen B, Weregemere NA, Noel H, Tshapenda GP, Mossoko M, Nsio J, et al. Urban yellow fever outbreak-Democratic Republic of the Congo, 2016: Towards more rapid case detection. PLoS Negl Trop Dis. 2018;12(12):e0007029. doi: 10.1371/journal.pntd.0007029 30532188 PMC6300298

[3] Gaythorpe KA, Hamlet A, Jean K, Garkauskas Ramos D, Cibrelus L, Garske T, et al. The global burden of yellow fever. Elife. 2021;10:e64670. doi: 10.7554/eLife.64670 33722340 PMC7963473

[4] Lindsey NP, Horton J, Barrett ADT, Demanou M, Monath TP, Tomori O, et al. Yellow fever resurgence: An avoidable crisis?. NPJ Vaccines. 2022;7(1):137. doi: 10.1038/s41541-022-00552-3 36323723 PMC9629880

[5] Yellow fever – African Region (AFRO). n.d. https://www.who.int/emergencies/disease-outbreak-news/item/2024-DON510

[6] Judson SD, Kenu E, Fuller T, Asiedu-Bekoe F, Biritwum-Nyarko A, Schroeder LF, et al. Yellow fever in Ghana: Predicting emergence and ecology from historical outbreaks. PLOS Glob Public Health. 2024;4(10):e0003337. doi: 10.1371/journal.pgph.0003337 39432459 PMC11493279

[7] Bonney JHK, Sanders T, Pratt D, Agbodzi B, Laryea D, Agyeman NKF, et al. Molecular Characterization of Circulating Yellow Fever Viruses from Outbreak in Ghana, 2021-2022. Emerg Infect Dis. 2023;29(9):1818–26. doi: 10.3201/eid2909.221671 37610174 PMC10461649

[8] World Health Organization. Disease Outbreak News; Yellow Fever - Ghana. 2021. https://www.who.int/emergencies/disease-outbreak-news/item/yellow-fever---ghana

[9] Inusah A-W, Collins G, Dzomeku P, Head M, Ziblim S-D. Knowledge, attitudes and practice towards yellow fever among nomadic populations: A cross-sectional study in yellow fever outbreak communities in Ghana. PLOS Glob Public Health. 2023;3(3):e0000733. doi: 10.1371/journal.pgph.0000733 36962969 PMC10019665

[10] World Health Organization. A global strategy to eliminate yellow fever epidemics (EYE) 2017–2026. Geneva: World Health Organization. 2018. https://apps.who.int/iris/handle/10665/272408

[11] World Health Organization. Laboratory manual for yellow fever. n.d. https://www.who.int/publications/i/item/9789240084476

[12] Eliminate yellow fever epidemics (EYE) strategy 2017-2026. World Health Organization. https://www.who.int/initiatives/eye-strategy

[13] Johnson BW, Demanou M, Fall G, Betoulle J-L, Obiekea C, Basile AJ, et al. Laboratory capacity assessments in 25 African countries at high risk of yellow fever, August-December 2018. Pan Afr Med J. 2021;38:402. doi: 10.11604/pamj.2021.38.402.28886 34381546 PMC8325472

[14] How improved yellow fever diagnostics are transforming management of the disease. n.d. https://www.gavi.org/vaccineswork/improving-yellow-fever-diagnostic-testing-more-efficient-effective-and-equitable

[15] Yamba EI, Aryee JNA, Quansah E, Davies P, Wemegah CS, Osei MA, et al. Revisiting the agro-climatic zones of Ghana: A re-classification in conformity with climate change and variability. PLOS Clim. 2023;2(1):e0000023. doi: 10.1371/journal.pclm.0000023

[16] Ghana Statistical Services. Multidimensional Poverty Ghana Report. n.d. https://statsghana.gov.gh/searchread.php?searchfound=NzcwODkzMzg5MTkuNzU3/search/qn815sn17q

[17] Basile AJ, Goodman C, Horiuchi K, Laven J, Panella AJ, Kosoy O, et al. Development and validation of an ELISA kit (YF MAC-HD) to detect IgM to yellow fever virus. J Virol Methods. 2015;225:41–8. doi: 10.1016/j.jviromet.2015.08.025 26342907 PMC4625539

[18] Domingo C, Charrel RN, Schmidt-Chanasit J, Zeller H, Reusken C. Yellow fever in the diagnostics laboratory. Emerg Microbes Infect. 2018;7(1):129. doi: 10.1038/s41426-018-0128-8 30002363 PMC6043483

[19] Timeliness of yellow fever specimen collection and transport in Ghana, 2018-2022. figshare. 2025. doi: 10.6084/m9.figshare.29265026.v2 PMC12716688 41417847

[20] RCore Team. R: A Language and Environment for Statistical Computing. Vienna, Austria: R Foundation for Statistical Computing. 2024. https://www.R-project.org/

[21] Wanyana MW, King P, Migisha R, Kwesiga B, Okello PE, Kadobera D, et al. Evaluation of the sentinel yellow fever surveillance system in Uganda, 2017-2022: strengths and weaknesses. BMC Infect Dis. 2024;24(1):686. doi: 10.1186/s12879-024-09580-x 38982363 PMC11234539

[22] Rachas A, Nakouné E, Bouscaillou J, Paireau J, Selekon B, Senekian D, et al. Timeliness of yellow fever surveillance, Central African Republic. Emerg Infect Dis. 2014;20(6):1004–8. doi: 10.3201/eid2006.130671 24857597 PMC4036780

[23] Nkrumah C, Forson PK, Nkrumah B, Owusu R, Musah MAA, Ahiataku DE, et al. Improving the specimen referral system in Ghana: findings from a landscape assessment. Front Public Health. 2025;13:1645873. doi: 10.3389/fpubh.2025.1645873 40963654 PMC12436456

[24] Nachega JB, Nsanzimana S, Rawat A, Wilson LA, Rosenthal PJ, Siedner MJ, et al. Advancing detection and response capacities for emerging and re-emerging pathogens in Africa. Lancet Infect Dis. 2023;23(5):e185–9. doi: 10.1016/S1473-3099(22)00723-X 36563700 PMC10023168

[25] Suu-Ire RD, Obodai E, Bonney JHK, Bel-Nono SO, Ampofo W, Kelly TR. Viral Zoonoses of National Importance in Ghana: Advancements and Opportunities for Enhancing Capacities for Early Detection and Response. J Trop Med. 2021;2021:8938530. doi: 10.1155/2021/8938530 33574853 PMC7860970

[26] Williams GS, Impouma B, Mboussou F, Lee TM-H, Ogundiran O, Okot C, et al. Implementing epidemic intelligence in the WHO African region for early detection and response to acute public health events. Epidemiol Infect. 2021;149:e261. doi: 10.1017/S095026882100114X 33985609 PMC8727712

[27] Chênes C, Albert H, Kao K, Ray N. Use of Physical Accessibility Modelling in Diagnostic Network Optimization: A Review. Diagnostics (Basel). 2022;12(1):103. doi: 10.3390/diagnostics12010103 35054270 PMC8774366

[28] 28.7-1-7: Rapid improvement for early disease detection and response. Resolve to Save Lives. n.d. https://resolvetosavelives.org/resources/introduction-to-7-1-7/

[29] Davis EH, Velez JO, Russell BJ, Basile AJ, Brault AC, Hughes HR. Evaluation of Whatman FTA cards for the preservation of yellow fever virus RNA for use in molecular diagnostics. PLoS Negl Trop Dis. 2022;16(6):e0010487. doi: 10.1371/journal.pntd.0010487 35704565 PMC9200311

[30] Kassambara H, Nana ML, Samassa F, Traoré MD. Sample Transport Optimization: Mali Pilot Study. Health Secur. 2020;18(S1):S92–7. doi: 10.1089/hs.2019.0061 32004128

[31] Ofosu-Appiah LH, Amelor DK, Ayensu B, Akyereko E, Rabiwu NI, Opare D, et al. An evaluation of the diagnostic performance characteristics of the Yellow Fever IgM immunochromatographic rapid diagnostic test kit from SD Biosensor in Ghana. PLoS One. 2022;17(1):e0262312. doi: 10.1371/journal.pone.0262312 34995319 PMC8741057

[32] Mobile labs deliver faster yellow fever test results. n.d. https://www.who.int/news-room/feature-stories/detail/mobile-labs-deliver-faster-yellow-fever-test-results

