# Supplementary material for: Timeliness of yellow fever specimen collection and transport in Ghana, 2018–2022
Source: PLOS Glob Public Health. 2025 Dec 19;5(12):e0005703. doi: 10.1371/journal.pgph.0005703 (PMC12716688; doi:10.1371/journal.pgph.0005703)
Supplement: S1 Fig — The 260 districts and 16 regions in Ghana that were analyzed for yellow fever testing from 2018-2022 are shown with the National Public Health and Reference Laboratory (NPHRL). The shapefile layers used to create the map were obtained from the Ghana Statistical Service (GSS)/Humanitarian Data Exchange (https://data.humdata.org/dataset/cod-ab-gha) and are publicly available under a CC BY-IGO license (https://data.humdata.org/faqs/licenses). (DOCX) [file pgph.0005703.s002.docx]

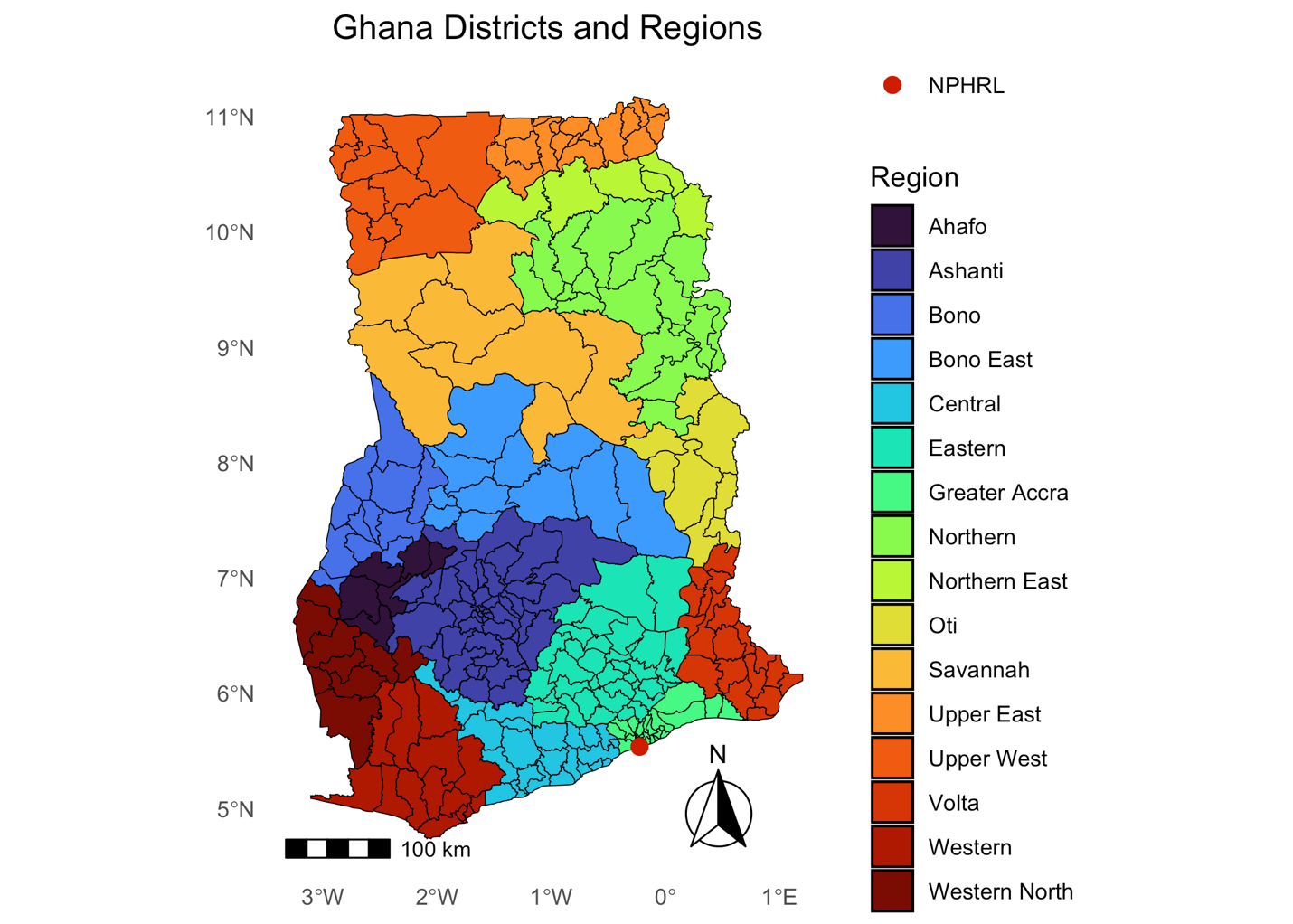


**S1 Fig. Ghana Districts and Regions 2018-2022**

The 260 districts and 16 regions in Ghana that were analyzed for yellow fever testing from 2018-2022 are shown with the National Public Health and Reference Laboratory (NPHRL). The shapefile layers used to create the map were obtained from the Ghana Statistical Service (GSS)/Humanitarian Data Exchange (<https://data.humdata.org/dataset/cod-ab-gha>) and are publicly available under a CC BY-IGO license (<https://data.humdata.org/faqs/licenses>).
